# Conflict Management in Nursing: Analyzing Styles, Strategies, and Influencing Factors: A Systematic Review

**DOI:** 10.3390/nursrep14040304

**Published:** 2024-12-23

**Authors:** Monica Nikitara, Mutu Roxane Dimalibot, Evangelos Latzourakis, Costas S. Constantinou

**Affiliations:** 1 Department of Health Sciences, School of Life and Health Sciences, University of Nicosia, Nicosia 1700, Cyprus; dimalibot.r@live.unic.ac.cy (M.R.D.); latzourakis.e@unic.ac.cy (E.L.); 2 Department of Basic and Clinical Sciences, Medical School, University of Nicosia, Nicosia 1700, Cyprus

**Keywords:** nurses, conflicts, strategies, management, styles, factors

## Abstract

Objective: This systematic review aimed to identify the most prevalent conflict management styles and strategies employed by nurses in clinical settings and to examine the factors associated with their selection. Methods: A comprehensive literature search was conducted following the PRISMA guidelines. Databases searched included PUBMED, CINAHL, Medline, and ProQuest, focusing on articles published between 2014 and 2024. Inclusion criteria were primary data studies involving nurses, published in English. The search strategy utilized Boolean operators to combine keywords related to nursing, conflict management, and healthcare settings. A total of 174 articles were initially identified, with 22 meeting the inclusion criteria after screening. The quality of the included studies was assessed using the Joanna Briggs Institute Qualitative Assessment and Review Instrument Critical Appraisal Checklist. The results were synthesized using content analysis. Results: The main findings from the 22 articles reviewed indicate that accommodation and collaboration/integration are the most common conflict management styles and strategies among nurses, with compromising also frequently employed. Factors such as age, experience, educational level, and workplace culture significantly influence the choice of conflict management strategies. Discussion: Nurses employ a variety of conflict management strategies depending on the context, individual preferences, and situational factors. Effective conflict resolution is closely linked to collaboration and communication, with proactive strategies being more effective in preventing conflicts. The findings underscore the need for tailored conflict management training to enhance job satisfaction and work relations in nursing environments. We acknowledge several limitations that may affect the interpretation and generalizability of our findings such as the diversity of the tools and the methodologies used by the included studies.

## 1. Introduction

Conflicts among healthcare professionals are prevalent in hospital settings, arising from differing values between physicians, nurses, patients, and their families. These conflicts occur both intra-professionally and inter-professionally [1]. Research indicates that healthcare professionals frequently face conflicts in their work environments. A quantitative study conducted among 128 nurses in Jordanian private and public hospitals revealed that nurses generally experience moderate levels of conflict, primarily in the form of intra-group disputes and disruptions caused by physicians [2]. Similarly, in the United States, conflicts are reported on a weekly basis, with approximately 20% of resident doctors experiencing serious conflicts with other staff, and nearly half of surgeons reporting disagreements concerning postoperative care goals [3].

Contrary to the common perception that conflict is often viewed negatively, it can also produce positive aspects if handled correctly. Conflict can contribute to the development of team-building skills, reflective thinking, the generation of new ideas, and the development of alternative solutions [4]. Conflict management is an important skill for leaders, as it can significantly contribute to the success of the teams, groups, units, or employees they oversee [5]. However, team conflicts can disrupt team dynamics and communication, reduce trust and performance, and negatively impact professionals’ mental health. Such conflicts might divert healthcare professionals’ focus from patient care and deplete their resources, jeopardizing team safety and the overall quality of patient care [6,7,8]. Concerning negative effects on nurses, these include, among others, the reduction in nursing performance and the increase in absenteeism and burnout [9,10,11]. On the other hand, the management of conflict in a positive manner has the potential to lead to the emergence of fresh ideas and new approaches, which can significantly boost morale and foster a heightened level of commitment among nurses and structural empowerment as well as improve their performance within the organization [12,13]. Research findings reveal that nursing professionals preferred to adopt constructive/positive conflict management styles instead of destructive/negative conflict management styles [14]. In this regard, the appropriate management of conflicts within a nursing context is linked with a proper provision of quality care, whereas in the opposite case, is associated with the provision of poor care to the patient, as a condition that not only affects patients but also the nurses as well as the whole organization [15,16].

Conflict is essentially a clash of opinions or arguments among individuals that may have the potential to harm an organization. In the context of a workplace, conflict typically occurs due to differences in personal agendas, perspectives, or goals compared to those of the whole team of an organization [17]. In this respect, some organizations, through mainly their leaders, seek to apply particular conflict management methods to address and resolve these disagreements in a way that brings positive outcomes for everyone involved while also benefiting the whole organization [18].

Therefore, managers need to acknowledge and understand the nuanced sources of conflict within this complex environment, to effectively address and manage the challenges that may arise while providing healthcare services. Consequently, the obtainment of the necessary skills to handle conflict is crucial for minimizing and effectively addressing the adverse outcomes it may bring about. The existing literature identifies five distinct styles regarding conflict management, that is, dominating (overwhelming the other party through decision-making or a position), obliging (addressing the requirements and concerns of the other parties), avoiding (purposefully neglecting a conflict), compromising (both sides compromise to achieve an agreement), and integrating (seeking an innovative resolution to a conflict that aims to meet the interests of both parties) [5]. Moreover, there are also five conflict management strategies that nurses could employ when in conflict situations, very similar to conflict management styles: competing (winning the dispute above all else), accommodating (allowing the other party to address their concerns), avoiding (ignoring the conflict), compromising (seeking a solution that partially satisfies both parties), and collaborating (find a solution that is mutually beneficial by working together) [14]. We chose to focus on these styles because they are widely recognized and frequently cited in the conflict management literature. These styles and strategies are foundational models in understanding conflict dynamics across various settings, providing a comprehensive framework for analyzing conflict management in nursing contexts. While other styles and strategies exist, these five are particularly relevant to the objectives of this study. Findings from the respective research show a wide range of strategies and practices employed by managers for conflict management, such as the findings that nursing faculty has a vital role in educating, preparing, and displaying constructive conflict resolution styles in nursing students [19], that introducing educational programs focusing on mediation and negotiation styles for conflict resolution proved to be beneficial for head nurses [20], and that nurses who encounter conflicts of moderate to high intensity tend to gravitate towards constructive rather than destructive management styles, yielding in this way more favorable outcomes for both themselves as well as for their health organizations [21].

The above emphasizes the influential impact of educational initiatives and faculty guidance in shaping the conflict resolution skills of nursing professionals. The implementation of targeted programs and the cultivation of constructive approaches have the potential to significantly enhance conflict resolution competencies among nursing students and practitioners alike. However, an unresolved issue that seems to emerge is that these styles are complicated, and for this reason, while a practitioner may employ one style more frequently than others, the selection of an approach can be influenced by personal traits, contextual elements, organizational settings, as well as relational dynamics [13].

Most literature reviews on nurses’ conflict management styles have primarily focused on the impact of these styles on the work environment. There is a lack of systematic reviews addressing the factors influencing the selection of specific management styles. It is therefore crucial to identify, based on the existing literature, the factors that guide nurses’ choice of conflict management styles and to highlight any gaps that may hinder their managerial effectiveness. This systematic review sought to identify the most prevalent conflict management styles and strategies employed by nurses in clinical settings and to examine the factors associated with their selection.

## 2. Methodology

A comprehensive systematic literature search was conducted following the recommendations outlined in the Preferred Reporting Items for Systematic Reviews and Meta-Analyses (PRISMA) statement, ensuring a thorough and systematic review was carried out [22].

### 2.1. Inclusion/Exclusion Criteria

Regarding the purposes of the current study, the following inclusion criteria were applied (Table 1).

The decision to concentrate on articles published between 2014 and 2024 was based on the need to ensure the relevance and contemporaneity of the findings. By focusing on the last decade, this review includes studies that account for recent developments and allows for the inclusion of a robust body of work, providing a comprehensive view of the conflict management literature while maintaining a manageable scope for analysis.

### 2.2. Information Sources and Search Strategy

The databases that were used to find the articles were the following: PUBMED, CINAHL, Medline, and ProQuest.

The search approach employed the Boolean operator OR between the keywords “nurses”, “conflicts”, “strategies” “styles”, “factors”, and “management” and comparable MeSH phrases. To narrow the search, phrases with different meanings were combined using the Boolean operator AND. The search strategy employed on the EBSCO platform for the specified databases is detailed in Table 2. We restricted the search to English-language journal articles with full-text access. However, many studies were excluded because they focused on health professionals other than nurses and healthcare settings other than nursing environments.

### 2.3. Study Selection Process

**Figure 1:**
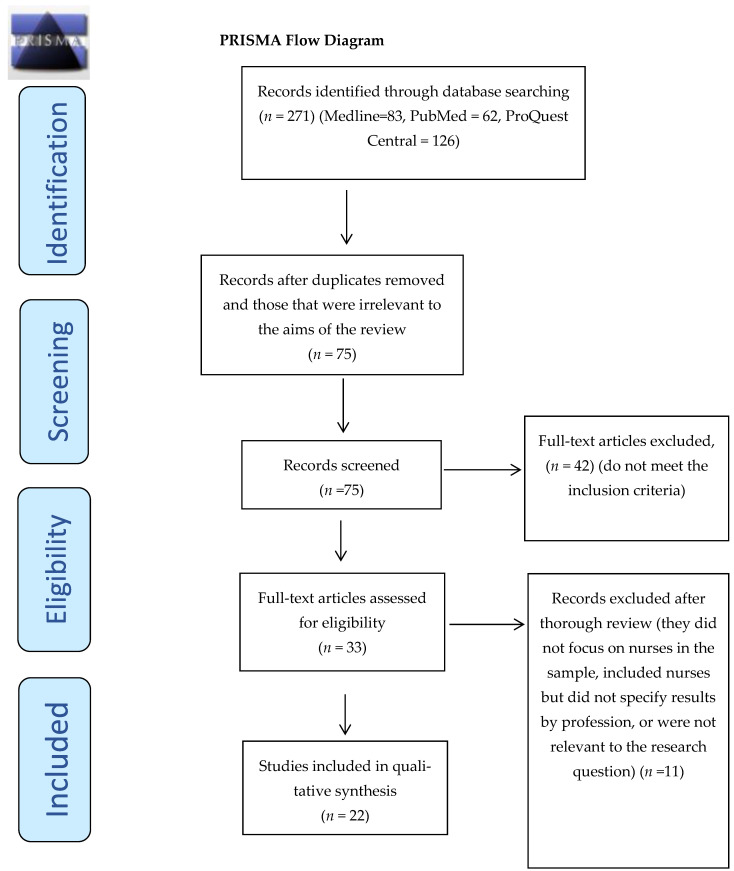
PRISMA flowchart with the search strategy of the systematic review.

The initial exploration yielded 174 articles regarding conflict management styles and strategies in a nursing context. After eliminating duplications, 75 articles underwent advanced screening. Of these articles, 42 studies were excluded as they did not pertain to the actual topic investigated. Subsequently, an additional 20 articles failed to meet the inclusion criteria. Consequently, only 22 articles ultimately met the stringent inclusion criteria developed in the current study. The PRISMA flow diagram shows in a detailed way the steps followed until the final selection of studies (Figure 1).

### 2.4. Data Collection Process

Two researchers independently gathered data from the chosen studies, extracting components, items, statements, or competencies that had achieved an expert consensus in the final round of each study. They specifically collected the following details: study title, authors’ names, publication year, aim, methodology, study design, and a summary of the main findings and results. After data extraction, the researchers meticulously reviewed the information multiple times, and then they coded and identified overarching themes.

### 2.5. Synthesis Methods

The data were analyzed using content analysis, which organized the findings into themes. Initially, a set of codes was created by carefully examining the results and findings section of a selected article, and these codes were refined as additional articles were reviewed. Each line of text was coded, and a code tree was used to identify emerging themes. Sub-themes were derived and combined from the interpreted meanings, undergoing further analysis until condensed into a single overarching theme. Content analysis aids in identifying and summarizing key elements within extensive data during the review process [23]. Themes related to conflict management styles and strategies were organized following the content analysis method suggested by Zhang and Wildemuth [23]. To ensure the validity of the results, a two-level quality assurance process was implemented. The authors independently conducted the review procedure, which included coding, categorizing, revisiting studies, and refining codes and categories. They then convened to discuss, refine the analysis, and finalize the results.

## 3. Results

This review adhered to the PRISMA guidelines (Supplement Table S1) [22], which provide a thorough and systematic method for conducting reviews and meta-analyses.

### 3.1. Study Selection

The initial search process yielded 174 articles related to nurses’ conflict management styles and conflict strategies. After removing duplicates and irrelevant articles through double-checking (Figure 1), 75 articles were included for advanced screening. Of these, 42 articles did not exclusively pertain to the nurses’ working environment. Two researchers independently reviewed the remaining 32 articles thoroughly. From this review, 10 articles were excluded as they did not meet the inclusion criteria. Ultimately, 22 articles met the criteria for inclusion. Further details about the included articles are provided in Table 3 below.

### 3.2. Study Characteristics

These 22 articles examine conflict management styles and resolution strategies used by nurses in various countries. Most of the studies used a descriptive correlational and cross-sectional design [1,2,3,4,5,6,7,8,9,10,11,12,13,14,16,17,19,20,21,22], while only two employed a qualitative approach [15,18]. Most of the studies [14] concern the process of conflict management styles, while the other nine studies refer to the conflict management strategies employed by nurses. Most of the studies come from countries of the Arab world, and their methodology is mainly of a cross-sectional design, with most of them using tools like ‘Rahim Organizational Conflict Inventory II [ROCI-II]’ and ‘Thomas–Kilmann Conflict Mode Instrument [TKI]’. Further details about the articles, including the author, year, tools, methodology, sample, and main results, are provided in Appendix A.

### 3.3. Study Assessment

The quality of the articles included in this review was evaluated using the Joanna Briggs Institute Qualitative Assessment and Review Instrument Critical Appraisal Checklist. This checklist assesses the methodological quality of a study and identifies potential biases in its conduct, design, and analysis. As shown in Appendix A, the studies included in the review primarily utilized descriptive correlational and cross-sectional designs [1,2,3,4,5,6,7,8,9,10,11,12,13,14,16,17,19,20,21,22], with two studies employing a qualitative approach [15,18]. All the included studies adhered to the Joanna Briggs criteria, providing comprehensive and detailed descriptions of their methodologies and procedures, as detailed in Table 3 and Table 4.

## 4. Results of Synthesis

Two major themes emerged from the data analysis, with sub-themes effectively addressing the research questions and highlighting the complex nature of the topic under investigation. The identified themes were as follows:

Theme 1: Conflict Management Styles in Nursing

- Prevalent conflict management styles;
- Influences on the chosen conflict management styles.

Theme 2: Conflict Management Strategies in Nursing

- Common strategies for conflict resolution;
- Conflict sources and resolution preferences.

Theme 1: Conflict Management Styles in Nursing

The studies reviewed provide a comprehensive overview of the conflict management styles employed by nursing professionals across various settings and demographics.

Subtheme: Prevalent Conflict Management Styles

Five common conflict management styles were identified in this study with accommodation and collaboration the most frequently used by nurses, followed by compromising and avoiding. The least common style is competing, which is the least preferred across most studies. Another style, the integrating style, has been reported but is not commonly observed in most studies.

### 4.1. Accommodation

Accommodation is one of the most prevalent conflict management styles among nurses [26,28,29,32,33]. Thomas (2015) found that accommodation was the most frequently adopted style with a mean score of 7.17, constituting 60% of the total score, particularly among nurses aged over 44 and 25–34 years [26]. This finding suggests that age and possibly the degree of experience within different age groups may play a significant role in determining conflict management preferences. One possible explanation for this trend is that nurses over 44 years old, who likely have more professional experience, may prefer accommodation as it allows for maintaining harmony and avoiding conflict escalation, which can be perceived as vital in sustaining long-term professional relationships and ensuring team cohesion. Their extensive experience might have shown them the benefits of choosing their battles and focusing on preserving collaboration and a positive work environment. Conversely, younger nurses in the 25–34 age bracket might opt for accommodation as they may still be establishing their professional identity and relationships within the workplace. Maharjan and Shakya (2021) also observed that newly recruited nurses predominantly used accommodating and collaborating styles, with a smaller percentage employing compromising, competing, and avoiding styles [29]. This trend suggests that accommodating and collaborating are common strategies among nurses, regardless of their experience level. Sharma et al. (2021) found that 34.7% of nurses reported using the accommodating style [26]. According to Maharjan and Shakya (2021), the accommodation style was the third most preferred, with a mean score of 3.55 [29]. Ahanchian et al. (2015) [28] found that accommodation had a mean score of 3.13, and Delak and Sirok (2022) [33] reported that the accommodation style accounted for 7.7% of conflict resolution styles.

### 4.2. Collaboration

The second most prevalent style is collaboration. Maharjan and Shakya (2021) identified collaboration as the most preferred style with a mean score of 4.17, followed by compromising and accommodating [29]. Assi and Eshah (2023) also found that the integrating (collaborating) approach was the most frequently adopted style [34]. Sharma et al. (2021) observed that newly recruited nurses predominantly used accommodating and collaborating styles, with 30.3% of nurses choosing the collaborating style [26]. In the study by Ahanchian et al. (2015), collaboration had a mean score of 3.95 [28]. Dewi et al. (2022) noted that nurses expressed a positive perception of head nurses who used the collaborating style, with a high percentage score of 85.75% [43].

### 4.3. Compromising

Compromising was a less chosen style throughout the studies. In Maharjan and Shakya (2021), compromising was the second most preferred style, with a mean score of 3.70 [29]. Sharma et al. (2021) found that only 18.9% of nurses employed the compromising style [26]. Ahanchian et al. (2015) reported a mean score of 3.46 for compromising, and Delak and Sirok (2022) found it accounted for 44.3% of conflict resolution styles [28,33]. Additionally, Mitlon et al. (2015) showed practical and statistical associations between the compromising style and colleague support [25].

### 4.4. Avoidance

Avoidance was among the least chosen styles. In Girish (2016), 36.7% of nurses employed the avoidance style, while in Sharma et al. (2021), only 5.4% adopted it [26,36]. Ardalan et al. (2017) found that avoidance had a mean score of 3.78 [30]. Johansen and Cadmus (2016) reported that 28% of nurses used the avoidant style, and Delak and Sirok (2022) found it was 42.3% [27,33]. Assi and Eshah (2023) noted that avoidance was the least employed style [34]. Al-Hamdan (2014) found that male nurses scored higher in the avoiding style compared to female nurses [24]. The preference for using the avoidance style among nurses may be influenced by factors such as gender differences, the organizational culture of the healthcare setting, experience levels, situational context, and perceived efficacy in specific scenarios.

### 4.5. Competing

Competing was the least preferred style across most studies. Thomas (2015) found that competing had the lowest mean score of 4.94, equivalent to 41% of participants, while Sharma et al. (2021) reported that only 10.7% of nurses leaned towards the competing style [26,32]. According to Maharjan and Shakya (2021), competing was the least preferred style, with a mean score of 3.06. Ahanchian et al. (2015) reported a mean score of 2.81 for competing, and Delak and Sirok (2022) found that competing accounted for only 2.3% of conflict resolution styles [28,29,33].

### 4.6. Integrating

The integrating style was not reported in most studies, but when it was included, it scored high. For example, Johansen and Cadmus (2016) found that 65% of nurses reported using integrating and obliging styles [27]. Assi and Eshah (2023) also found the integrating approach to be the most frequently adopted style [34]. Al-Hamdan (2014) found that female nurses scored higher in the integrating style compared to male nurses [24].

#### 4.6.1. Influences on the Chosen Conflict Management Styles

Girish (2016) and Al-Hamdan (2014) explored the influence of demographic variables on conflict management styles [24,36]. Girish found no significant association between demographic factors and preferred conflict resolution approaches, indicating that age, gender, and other variables did not significantly impact the choice of conflict management styles among the surveyed nursing staff. In contrast, Al-Hamdan (2014) identified gender as a significant factor, with female nurses scoring higher in integrating and lower in avoiding styles compared to their male counterparts [24]. Additionally, the type of hospital influenced the use of integrating and compromising styles, with government hospital nurses being less inclined to use these styles. Mitlon et al. (2015) examined the correlation between conflict management styles and job demands and resources [25]. They found that the integrating style was associated with colleague support, time demands, and feedback, while the dominating style was linked to nurses’ wages. The compromising style was also associated with colleague support. These findings highlight the importance of job characteristics in shaping conflict management preferences.

Ardalan et al. (2017) and Assi and Eshah (2023) provided insights into the perceptions of conflict and the role of emotional intelligence [30,34]. Ardalan found that nurses perceived moderate levels of conflict in their workplace and primarily employed controlling, avoiding, and resolving conflict styles. Assi and Eshah (2023) identified a significant association between higher levels of emotional intelligence and the use of integrating, obliging, and compromising styles, suggesting that emotional intelligence plays a crucial role in effective conflict management [34].

Johansen and Cadmus (2016) explored the impact of conflict management styles and a supportive work environment on work-related stress among emergency department nurses [27]. They found that the avoidant style was associated with lower levels of work stress, while a supportive work environment and the avoidant style were significant predictors of work stress. This study underscores the importance of a supportive work environment in mitigating stress and promoting effective conflict management. Ariyalakshmi and Mahalakshmi (2019) examined conflict management styles among nursing students [35]. They found that most students favored the approach style, with a smaller percentage choosing avoidance. No significant associations were found between demographic variables and conflict management styles, indicating that these factors did not significantly influence the students’ preferences.

In summary, the studies collectively indicate that accommodation and collaboration are common conflict management styles among nurses, with demographic factors such as age and gender influencing these preferences. Job demands and resources, emotional intelligence, and a supportive work environment also play crucial roles in shaping conflict management strategies. Effective conflict resolution is closely linked to collaboration and communication, with proactive strategies being more effective in preventing conflicts. These findings underscore the need for tailored conflict management training that considers demographic and contextual factors to enhance job satisfaction and work relations in nursing environments.

#### 4.6.2. Theme 2: Conflict Management Strategies in Nursing

The studies reviewed provide a comprehensive overview of conflict management strategies employed by nurses across various settings and demographics.

#### 4.6.3. Common Strategies for Conflict Resolution

The findings from the studies by Lahana et al. (2017), Baddar et al. (2016), Bashir et al. (2022), Sbordoni et al. (2020), and Ovčina et al. (2023) provide valuable insights into the conflict resolution strategies employed by nurses and the demographic influences on these strategies [37,38,39,41,44]. Lahana et al. (2017) and Baddar et al. (2016) both highlight avoidance and collaboration as predominant conflict management styles among nurses [38,39]. Lahana et al. found that avoidance was the most common strategy, particularly among experienced and managerial nurses, while collaboration was favored by highly educated nurses. Similarly, Baddar et al. noted that younger nurses (under 30) preferred compromising and accommodating strategies, whereas older nurses (over 30) leaned towards collaboration. Bashir et al. (2022) also observed that younger nurses (21–35 years) predominantly used compromising strategies, while older nurses (36–65 years) favored avoiding [37]. This trend suggests that age and experience significantly influence the choice of conflict management strategies, with younger nurses more inclined towards compromise and older nurses towards avoidance.

Sbordoni et al. (2020) highlighted the role of emotional intelligence, dialogue, and empathic listening in conflict mediation [44]. Identified conflict-generating sources included non-compliance with institutional standardization and lack of comprehension regarding the comprehensive role of nursing care. Ovčina et al. (2023) found that nurses commonly employ strategies such as exchanging information and opinions, engaging in negotiations, and actively seeking concessions to resolve conflicts [41].

#### 4.6.4. Conflict Sources and Resolution Preferences

Aljuaid and Alkarani (2023) identified two main themes: conflicts arising from a lack of awareness regarding work policies and communication breakdowns [45]. They emphasized pre-emptive strategies such as identifying conflict sources in advance, conducting regular meetings, and increasing awareness of policies to mitigate conflicts. This proactive approach contrasts with the more reactive strategies observed in other studies. Başoğul (2020) found a positive correlation between conflict management strategies and teamwork attitudes [40]. Compromising was the most frequently employed strategy, followed by integrating and obliging. This study underscores the importance of teamwork and communication in effective conflict resolution.

#### 4.6.5. Gender and Cultural Differences

Hussain et al. (2023) highlighted gender-based differences in conflict resolution preferences [31]. Female nurses were more inclined towards compromising, while male nurses showed a lesser tendency to avoid conflict. This study also noted that younger nurses (20–30 years) preferred accommodating strategies, indicating that both gender and age play crucial roles in conflict management. Akel and Elazeem (2015) compared the perspectives of nurses and physicians, revealing significant disparities in conflict causes and resolution strategies [42]. Physicians were more likely to adopt compromising strategies, while nurses favored accommodating. This difference underscores the varying approaches to conflict resolution between different healthcare professionals. These differences can be attributed not only to individual preferences or professional training but also to the differing levels of power and influence these groups hold within healthcare organizations. Physicians typically occupy positions of greater authority and decision-making power, which can empower them to engage in compromising strategies that require negotiation and balancing of interests. On the other hand, nurses, who often work under hierarchical constraints, may opt for accommodating strategies that reflect their position in the organizational structure and their role in maintaining harmony and patient care continuity. Sbordoni et al. (2020) and Dewi et al. (2022) provided qualitative insights into conflict management [43,44]. Sbordoni et al. emphasized the importance of emotional intelligence, dialogue, and empathic listening in conflict mediation [44]. Dewi et al. found that head nurses predominantly used compromising and collaborating strategies, which were positively perceived by their subordinates [43]. Conversely, avoiding strategies were viewed negatively.

In summary, avoidance and collaboration are common strategies, with younger nurses favoring compromise and older nurses leaning towards avoidance. The studies collectively indicate that conflict management strategies among nurses are influenced by demographic factors such as age, experience, and education. Gender differences also play a role, with female nurses more inclined towards compromising. Effective conflict resolution is closely linked to teamwork, communication, and emotional intelligence, with proactive strategies being more effective in preventing conflicts. These findings underscore the need for tailored conflict management training that considers demographic and cultural differences to enhance job satisfaction and work relations in nursing environments.

## 5. Discussion

As can be seen from the results, the terms “conflict management styles” and “conflict management strategies” are often used interchangeably, while in some cases have slightly different meanings depending on their context. For instance, from the above analysis of the studies, we can surmise that conflict management styles usually refer to the general approaches or attitudes that health professionals use to address conflicts. These styles are often characterized by behaviors, attitudes, and responses to conflict situations. On the other hand, conflict management strategies often concern the specific actions or procedures employed by nurses in their efforts to deal with conflicts within a particular context or situation. These strategies can involve, among others, communication methods, negotiation tactics, problem-solving approaches, and decision-making processes that seek to resolve conflicts in the best possible way. In the context of nursing, conflict management styles and strategies are closely related. Nurses may employ various conflict management styles and strategies or a combination of them, depending on their personal preferences, the nature of the conflict, the individuals involved, and the specific circumstances of the healthcare environment. To the above clarifications, the comparison and contrast of our findings with other related studies, as well as the limitations that emerged through the research process will be presented in a unified way for both conflict management styles and strategies.

Taking into consideration the above, the findings from this systematic literature review provide a comprehensive understanding of the conflict management styles and strategies employed by nurses across various settings and demographics. This review specifically identified five common conflict management styles among nurses, which they also used as conflict strategies: accommodation, collaboration, compromise, avoidance, and competition. Among these, accommodation and collaboration emerged as the most frequently used styles, while competing was the least preferred. These findings seem to be comparable largely to other studies. More specifically, according to Labrague et al. (2018) and Leveillee (2018), collaboration emerged as the most prevalent style utilized in managing conflicts among nursing professionals, with accommodation following closely behind [14,46]. Nursing researchers have established that collaborative and compromising approaches lead to effective conflict management, while avoidance, accommodating, and competing strategies tend to be ineffective [47,48]. The prevalence of accommodation as a conflict management style suggests that many nurses prioritize maintaining harmony and avoiding confrontation. This approach can be beneficial in preserving relationships but may also lead to unresolved issues if overused. The high adoption of this style among both newly recruited and experienced nurses indicates its perceived effectiveness in various contexts. On the other hand, collaboration is highly valued, particularly among highly educated nurses. This style promotes open communication and joint problem-solving, which can lead to more sustainable conflict resolution. The positive perception of head nurses who use collaboration underscores its importance in leadership roles. Compromising is also another strategy that is frequently employed, but mainly by younger nurses and those with less experience. This strategy involves finding middle ground and making concessions to reach a resolution that partially satisfies all parties involved. Moreover, accommodating, where nurses prioritize the concerns and needs of others over their own, was another strategy found in the reviewed studies. However, it is often employed to a lesser extent compared to avoidance, collaboration, and compromising. Finally, competing, though less common, is still present as a conflict management strategy among nurses. This approach refers to pursuing one’s own interests at the expense of others, as a strategy that often leads to a win–lose outcome. However, in the healthcare settings where collaboration and teamwork are emphasized, the competing strategy was found to be the least prevalent strategy due to its potential to escalate conflicts.

In addition, factors such as age, experience, educational level, and workplace culture influence the choice of conflict management strategies among nurses. Younger nurses and those with less experience may lean towards compromising or accommodating strategies, while more experienced nurses may prefer collaboration or avoidance due their familiarity with effective problem-solving practices and workplace dynamics. In summary, nurses employ a variety of conflict management strategies depending on the context of the conflict, individual preferences, and situational factors. These strategies play a crucial role regarding the development of effective communication, teamwork, and ultimately, the provision of quality patient care. Furthermore, the findings by another study seem to be harmonized with some of the external factors like setting, experience, and gender found in the current study, which affect nurses’ adoption and employment of particular styles and strategies, including personal characteristics (marriage, level of education, etc.), contextual factors (private or public hospitals), and interpersonal conditions (professional relationships with head nurses or physicians) [49]. Female nurses are more likely to use integrating and less likely to use avoiding styles compared to their male counterparts. Moreover, the importance of effective communication and emotional intelligence as shown in the current study are harmonized with the findings of the recent review by Kiyumi (2023), according to which the conflict resolution capabilities of healthcare leaders are notably impacted by their professional communication skills and level of emotional intelligence [50].

The findings underscore the need for tailored conflict management training that considers demographic and cultural differences. Such training can help nurses develop a range of conflict resolution strategies and choose the most appropriate approach based on the specific context and individuals involved. Additionally, fostering a supportive work environment can mitigate stress and promote effective conflict management.

## 6. Limitations

Like all other research, the current study had a number of limitations. To begin with, while this review encompassed research from various global regions, a notable limitation concerns the scarcity of studies that contextualized their findings within the cultural backgrounds of their respective countries, as an oversight that may impede the generalizability and applicability of the findings across diverse populations [14]. As LeBaron (2003) suggests, cultural influences are deeply embedded in every conflict scenario, as conflicts occur naturally within human relationships [51]. In this regard, the way in which individuals perceive relationships and navigate conflicts, which are inevitable in any interaction involving two or more people, is profoundly shaped by their cultural background. Research efforts accounting for this seem to be crucial for effectively managing the complexities that occur due the diversity that characterizes the current healthcare organizations, patient populations, nursing workforce, and the broader healthcare landscape.

Another limitation concerns the fact that while most of the studies in this review utilized well-established and standardized tools like the Rahim Organizational Conflict Inventory-II and the Thomas–Kilmann Conflict Mode Instrument, some studies (6 out of 23) employed alternative tools with different structures, contents, and item numbers. This diversity in tools produces several challenges in comparing, contrasting, and extrapolating findings across studies. Additionally, some studies did not report the reliability and validity of the instruments used. The absence of such vital information may have influenced the reliability and generalizability of the findings [14].

## 7. Conclusions

This study focused on conflict management styles and strategies among nurses, recognizing the inherent complexities within healthcare settings. Conflict, while often viewed negatively, can lead to positive outcomes if managed effectively. In the nursing profession, conflicts arise due to organizational complexities, diverse roles, communication challenges, and varied stakeholder interests. As the findings of the current literature review indicate, a spectrum of strategies and styles regarding conflict management are employed in healthcare settings. Through an amalgamation of diverse studies, several pivotal insights surface, enriching the comprehension of conflict resolution within nursing contexts.

Firstly, this review displays the repertoire of conflict management styles and strategies that are prevalent among nurses. Accommodation, collaboration, compromising, avoidance, and competing emerge as primary approaches, each employed in different circumstances and settings. This understanding adds to the adaptive nature of nurses’ responses to conflicts, highlighting the complexity of interpersonal dynamics in healthcare.

Moreover, this literature review elucidates the multifaceted influences that shape nurses’ conflict management choices. Demographic factors such as age, experience, and educational background, alongside contextual variables like workplace culture, have deep impacts on nurses’ strategic preferences. These insights underscore the need for tailored interventions and training initiatives that account for the diverse profiles and environments encountered in nursing practice.

Finally, this review offers various practical recommendations to advance conflict resolution processes in nursing. These practices concern the development of tailored training programs, the formulation of evidence-based guidelines, the cultivation of interdisciplinary collaboration, and the utilization of theoretical frameworks. By following these recommendations, stakeholders can foster an environment that is beneficial to constructive conflict resolution, simultaneously improving patient care outcomes and enhancing the nursing profession. By bridging empirical findings with theoretical frameworks and offering pragmatic recommendations, this review contributes to the cultivation of a culture of effective conflict resolution in healthcare. Ultimately, the efforts in this field should be harmonized with the principal goals of optimizing patient care outcomes and fostering a resilient and consistent nursing workforce.

## Figures and Tables

**Table 1:** Inclusion and exclusion criteria.

Inclusion Criteria	Exclusion Criteria
Articles with primary data	Secondary sources
The study sample comprised nurses	The sample did not include nurses
Published between 2014 and 2024	Published earlier than 2014
Written in English	Not written in the English language

**Table 2:** Search strategy PIC.

Population		Interest		Context
(TI (“Registered Nurs*” OR “RN” OR “Nurs*” OR “Nursing staff” OR “Clinical nurse” OR “Nurse specialist” OR “Nurse clinician” OR “Nursing care provider” OR “Nursing team member”) OR AB (“Registered Nurs*” OR “RN” OR “Nurs*” OR “Nursing staff” OR “Clinical nurse” OR “Nurse specialist” OR “Nurse clinician” OR “Nursing care provider” OR “Nursing team member”) OR DE “Nursing” OR MH “Nursing” OR “Nurses”)	and	(TI (“Conflict Management” OR “Strategies” OR “Resolution*” OR “Styles”) (“Factors” OR “Influences” OR “Determinants”) OR AB (“Conflict Management “ OR “ Strategies “ OR “ Resolution*” OR “Styles”) (“Factors” OR “Influences” OR “Determinants”) OR DE “Conflict Management” OR MH “Conflict Management” OR “Strategies” OR “Resolution”)	and	(TI (“Hospital” OR “Health Care Facilities” OR “Health Services” OR “Health Care” OR AB (“Hospital*” OR “Health Care Facilities” OR “Health Services” OR “Health Care”) OR DE “Hospital” OR MH “Hospital” OR “Health Care Facilities”))

**Table 3:** JBI Critical Appraisal Checklist for Analytical Cross-Sectional Studies.

Authors and Year	Q1	Q2	Q3	Q4	Q5	Q6	Q7	Q8
Al-Hamdan, Z., Norrie, P. and Anthony, D. (2014). [24]	√	√	√	√	√	√	√	√
Milton, D. R., Nel, J. A., Havenga, W. and Rabie, T. (2015) [25]	√	√	√	√	√	√	√	√
Sharma, T., Bhatia, M., Andrews, G. and Mahesh, R. (2021) [26]	√	√	√	√	√	√		√
Johansen, M.L. and Cadmus, E. (2016) [27]	√	√	√	√	√	√	√	√
Ahanchian, M.R., Emami Zeydi, A. and Armat, M.R. (2015) [28]	√	√	√	√	√	√	√	√
Maharjan, S. and Shakya, J. (2021) [29]	√	√	√	√	√	√	√	√
Ardalan, F., Valiee, R. and Valiee, S. (2017) [30]	√	√	√	√	√	√	√	√
Hussain, N., Kousar, R., Asif, M. and Bibi, S. (2023) [31]	√	√	√	√	√	√	√	√
Thomas, C. (2015) [32]	√	√	√	√	√	√	√	√
Delak, B. and Sirok, K. (2022). Physician–nurse conflict resolution styles in primary health care. [33]	√	√	√	√	√	√	√	√
Assi, M. D. and Eshah, N. F. (2023) [34]	√	√	√	√	√	√	√	√
Ariyalakshmi, B. and Mahalakshmi, H. (2019) [35]	√	√	√	√	√	√	√	√
Girish, V. H. (2016). [36]	√	√	√	√	√	√	√	√
Bashir, K., Shahzadi, A., Ashraf, A. and Bashir, N. (2022). [37]	√	√	√	√	√	√	√	√
Lahana, E., Tsaras, K., Kalaitzidou, A., Galanis, P., Kaitelidou, D. and Sarafis, P. (2017). [38]	√	√	√	√	√	√	√	√
Baddar, F., Salem, O. A. and Villagracia, H. N. (2016). [39]	√	√	√	√	√	√	√	√
Başoğul, C. (2020). [40]	√	√	√	√	√	√	√	√
Ovčina, A., Begić, S., Eminović, E., Hrapović, E., Marjanović, M., Dido, V., Konjo, H. and Šljivo, E. (2023). [41]	√	√	√	√	√	√	√	√
Akel, D. T. and Elazeem, H. A. (2015). [42]	√	√	√	√	√	√	√	√
Dewi, R. R. C., Yanti, N. E. D., Rahajeng, I. M. and Krisnawati, K. M. S. (2022). [43]	√	√	√	√	√	√	√	√

**Table 4:** Risk of bias assessed by the Joanna Briggs Institute Critical Appraisal Checklist for Qualitative Studies results.

Authors and Year	Q1	Q2	Q3	Q4	Q5	Q6	Q7	Q8	Q9	Q10
Sbordoni, E. de C., Madaloni, P. N., Oliveria, G. S. de., Fogliano, R. R. F., Neves, V. R. and Balsanelli, A. P. (2020) [44]	√	√	√	√	√	√	√	√	√	√
Aljuaid, J.A. and Alkarani, A.S. (2023) [45]	√	√	√	√	√	√	√	√	√	√

## Data Availability

The articles’ data supporting this systematic review are from previously reported studies and datasets, which have been cited. The processed data are available in Table 3 and the reference list. Further information can be requested from the corresponding author.

## Supplementary Materials

The following supporting information can be downloaded at: https://www.mdpi.com/article/10.3390/nursrep14040304/s1, Table S1: PRISMA 2020 Checklist.

## Public Involvement Statement

There was no public involvement in any aspect of this research.

## Guidelines and Standards Statement

This manuscript was drafted in accordance with the PRISMA2020 Guidelines [22] for systematic reviews.

## Appendix A

**Table A1:** Characteristics of the articles reviewed.

Authors/Title	Aims	Methodology	Sample
1. Al-Hamdan, Z., Norrie, P. and Anthony, D. (2014). Conflict management styles used by nurses in Jordan. *Journal of Research in Nursing,* 19(1), 40–53. [24]	The study aimed to investigate the conflict management styles employed by Jordanian nurses and their correlation with demographic variables such as age, gender, and educational level.	This study utilized a cross-sectional Rahim Organization Conflict Inventory (ROCI II) questionnaire.	The questionnaires were distributed to 420 nurses across three different types of hospitals in Amman. Three hundred and fifty nurses completed the questionnaire.
2. Milton, D. R., Nel, J. A., Havenga, W. and Rabie, T. (2015). Conflict management and job characteristics of nurses in South African public hospitals. *Journal of Psychology in Africa*, 25(4), 288–296. [25]	This study sought to evaluate the correlation between various conflict handling styles and different job demands and resources among nurses in multiple public hospitals in Gauteng, South Africa.	In this study, a cross-sectional survey design was used to collect the data. Rahim’s Organizational Conflict Inventory II [ROCI-II]	The study included 205 nurses. The majority of participants were female (87.3%), aged between 46 and 60 years (38.5%). Most participants were married (40.5%) and held a diploma or certificate (60%) in nursing. The largest portion of participants worked in the medical ward (24.9%) and were enrolled nurses (37.6%).
3. Sharma, T., Bhatia, M., Andrews, G. and Mahesh, R. (2021). Conflict management styles of nurses at All India Institute of Medical Sciences, New Delhi, India. Journal of Applied Sciences and Clinical Practice, 2, 59. [26]	The objective of the study was to identify the conflict-management styles employed by recently recruited nursing personnel at a tertiary care hospital.	In this study, a descriptive cross-sectional survey was conducted, where participants were given a questionnaire comprising two sections. Part I collected demographics. Part II utilized the Standardized Thomas–Kilmann Conflict Mode Instrument to assess the conflict management styles of nurses.	The study was conducted on 297 newly joined nurses working in a tertiary care hospital. The majority (97.4%) were aged between 20 and 30 years, while female nurses accounted for 71.7% of the participants. Approximately 40% of the nurses had 1–3 years of professional experience, while 37.3% had more than 3 years of experience.
4. Johansen, M.L. and Cadmus, E. (2016). Conflict management style, supportive work environments and the experience of work stress in emergency nurses. *Journal of Nursing Management,* 24(2), 211–218. [27]	The objective was to investigate the conflict management styles utilized by emergency department (ED) nurses for resolving conflicts and to ascertain whether their conflict management style and a supportive work environment influenced their experience regarding work-related stress.	This study employed a descriptive, correlational design. Data were gathered through a demographic questionnaire along with the expanded Nursing Work Stress Scale (ENSS), the Abbreviated Survey of Perceived Organizational Support (SPOS), and the Rahim Organizational Conflict Inventory-II (ROCI-II).	The final sample size for this study was 222 staff nurses who worked in hospital-based emergency departments.
5. Ahanchian, M.R., Emami Zeydi, A. and Armat, M.R. (2015). Conflict Management Styles Among Iranian Critical Care Nursing Staff: A Cross-sectional Study. *Dimensions of Critical Care Nursing,* 34(3), 140–145. [28]	The objective of this study was to assess the frequency of conflict management styles and their associated factors among critical care nursing staff in Iran.	This study utilized a descriptive cross-sectional design. The questionnaire comprised two sections. The first part collected demographic information. The second part included the Rahim Organizational Conflict Inventory II (ROCI II).	In this study, a total of 149 critical care nurses who worked in the critical care units of 4 teaching hospitals in Sari (Iran) were evaluated.
6. Maharjan, S. and Shakya, J. (2021). Conflict management styles among nurses at a teaching hospital in Chitwan. *Journal of Chitwan Medical College*, 11(38), 37–40. [29]	This study aimed to find the conflict management styles among nurses at a Teaching Hospital, in Chitwan.	A descriptive cross-sectional research design was used in this study. The data were collected through a set of structured self-administered questionnaire developed for sociodemographic and professional-related information, and the standardized tool of Rahim Organizational Conflict Inventory II (ROCI-II) was used.	The sample of 50 nurses were selected using the non-probability convenience sampling method and the data were collected from 6 December 2020 to 19 December 2020.
7. Ardalan, F., Valiee, R. and Valiee, S. (2017). The level of job conflicts and its management styles from the viewpoint of Iranian nurses. *Nursing Practice Today,* 4(1), 44–51. [30]	The aim was to evaluate the degree of job conflict experienced by nurses in their professional roles and to identify the prevailing management styles utilized to address conflicts.	The study employed a descriptive cross-sectional design. The study utilized the DuBrin Job Conflict Questionnaire, comprising 20 questions with options for often agreeing and often disagreeing. Additionally, the Putnam and Wilson Conflict Management Styles Questionnaire was employed to measure conflict management styles, which involves 30 questions encompassing the avoiding, resolving, and controlling styles.	The study was carried out with the participation of 423 employed nurses in different units of the educational hospitals of Kurdistan University of Medical Sciences, Sanandaj, Iran.
8. Hussain, N., Kousar, R., Asif, M. and Bibi, S. (2023). Conflict Resolution Styles among Nursing Staff Public Sector Hospital—A Cross Sectional Study. Annals of Punjab Medical College (APMC), 17(1), 50–53. [31]	The aim was to explore the various conflict management experiences encountered by nurses in their workplace environments.	This study employed a cross-sectional design. The questionnaire comprised two sections: the first section collected demographic data about the participants, while the second section focused on conflict resolution among nursing staff. The Thomas–Kilmann Conflict MODE Instrument was utilized.	A total of 197 nurses participated.
9. Thomas, C. (2015). Conflict resolution style used by nursing professionals. *International Journal of Advances in Nursing Management,* 3(3), 7–14. [32]	The objective of the study was to determine the primary conflict resolution strategies employed by nursing professionals in both clinical and academic settings.	The study utilized a descriptive cross-sectional survey approach. Data collection was conducted using a non-probability purposive sampling technique, and the Standardized Thomas–Kilmann Conflict Mode Instrument (TKI) was employed.	The data were collected from 100 nursing professionals who were working in both clinical and academic settings.
10. Delak, B. and Sirok, K. (2022). Physician–nurse conflict resolution styles in primary health care. *Nursing Open,* 9(2), 1077–1085. [33]	The aim of the study was to investigate the conflict resolution styles employed in collaborative interactions between physicians and nurses within the primary healthcare setting.	The study employed a descriptive, cross-sectional, correlational design. The instrument used was the validated Slovene translation of the Thomas–Kilmann Conflict MODE Instrument.	The study comprised 173 nurses (58.1%) and 125 physicians (41.9%). In terms of gender distribution, 8.1% were male and 91.9% were female. The vast majority had been working for more than 5 years (84.45%).
11. Assi, M. D. and Eshah, N. F. (2023). Emotional intelligence and conflict resolution styles among nurse managers: a cross-sectional study. *British Journal of Healthcare Management,* 29(6), 53–62. [34]	The main objective was to elucidate the role of emotional intelligence in aiding nurse managers to effectively managing conflicts within healthcare settings.	The study employed a cross-sectional and explanatory design, by integrating latent variables represented by the structural equation model (SEM). Various sociodemographic variables were taken into account; while conflict management styles were assessed using the Rahim Organizational Conflict Inventory-II (ROCI-II), the emotional intelligence was measured through the Rotterdam Emotional Intelligence Scale (REIS) and job satisfaction was evaluated using the S20/23 tool.	The participants consisted of a convenience sample of 197 nurse managers selected from five public hospitals and two university hospitals in Jordan.
12. Ariyalakshmi, B. and Mahalakshmi, H. (2019). A Study to Assess the Conflict Management Style Among the Nursing Students in Madha College of Nursing, Chennai. *International Journal of Community Health Nursing,* 1(1), 24–29. [35]	The aim was to assess the conflict management styles among nursing students and identify any associations between these styles and selected demographic variables.	This descriptive study involved the preparation of an opinion survey to evaluate the conflict management styles among students. The survey comprised 24 items focusing on various aspects of conflict management styles.	Of the 30 Indian students surveyed, the majority (86.7%) were in the age group below 23 years, while 70% of them were pursuing a bachelor’s degree.
13. Girish, V. H. (2016). A descriptive study to assess the conflict management style among staff nurses in multispecialty hospital, Health city campus, Bengaluru. *International Journal in Management and Social Science,* 4(6), 575–580. [36]	The aim was to assess the conflict management styles demonstrated by staff nurses and explore potential associations with selected demographic variables including age, gender, education, marital status, designation, monthly income, and institution changes.	This descriptive study selected participants through a non-probability convenient sampling technique. Data collection was conducted using a structured interview schedule, which had been validated by experts in the nursing faculty.	30 nurses.
14. Bashir, K., Shahzadi, A., Ashraf, A. and Bashir, N. (2022). Conflict Management Among Nurses in Tertiary Care Hospital Lahore: Conflict Management Among Nurses. Journal of Nursing & Midwifery Sciences), 2(02), 26–30. [37]	The aim of the study was to analyze the most frequently occurring conflict management strategy utilized by nurses in the hospital setting.	A descriptive cross-sectional study design was used to carry out this study. A random sampling method was used to collect data from nurses using a self-administered questionnaire.	Responses of 122 female nurses were recorded and analyzed in this study.
15. Sbordoni, E. de C., Madaloni, P. N., Oliveria, G. S. de., Fogliano, R. R. F., Neves, V. R. and Balsanelli, A. P. (2020). Strategies used by nurses for conflict mediation. Revista Brasileira De Enfermagem, 73, e20190894. [44]	The aim of the study was to understand what strategies are used by nurses to mediate conflicts.	This qualitative study was guided by the Consolidated Criteria for Reporting Qualitative Research (COREQ) and carried out through oral history. Data collection was carried out through semi-structured interviews by using a guiding question “What are the ways you use to mediate conflicts?”	Seven professionals participated in this research, four nurses and three nurses. Two of them worked the morning shift, one the afternoon, and four the two-night shifts.
16. Lahana, E., Tsaras, K., Kalaitzidou, A., Galanis, P., Kaitelidou, D. and Sarafis, P. (2017). Conflicts management in public sector nursing. International Journal of Healthcare Management, 12, 1–7. [38]	The aim of the study was to identify the main sources of interpersonal conflicts in nurses and their strategies in handling conflict individually as well as by nurse management.	A cross-sectional design was used for this study. A self-administered questionnaire was used in order to assess the existence of conflicts and their management in the public healthcare sector. The five-part questionnaire is based on the original questionnaire of Tenglilimoglu and Kisa, which is specific for conflicts in the hospital setting.	One hundred and forty-three questionnaires were handed out in the nursing and nursing assistant’s staff of the two nursing divisions of the pathology (41 nurses) and surgical (102 nurses) wards.
17. Baddar, F., Salem, O. A. and Villagracia, H. N. (2016). Conflict resolution strategies of nurses in a selected government tertiary hospital in the Kingdom of Saudi Arabia. *Journal of Nursing Education and Practice,* 6(5), 91–99. [39]	This research sought to explore the methods used by nurses in a Saudi Arabian hospital regarding the resolution of conflicts.	A descriptive correlational research design was utilized for this study, by focusing on all nurses employed in the chosen hospital as the target population. Sampling involved nurses currently working in Riyadh during the study period. The data-gathering instrument consisted of 20 items grouped into five sub-scales, adapted from Elliot (2010).	78 nurses.
18. Aljuaid, J.A. and Alkarani, A.S. (2023). Conflict among Saudi nurses in hospitals: a qualitative study. *Nursing Communication,* 7, e2023015. [45]	The study aimed to explore the sources of conflicts and examine how nurses in Saudi Arabia handle conflicts across three different hospitals.	A qualitative descriptive study was undertaken, by employing in-depth semi-structured interviews for data collection. The interview questions were crafted to prompt open-ended responses, enabling participants to offer comprehensive insights into their conflict experiences. Further inquiries were incorporated during the interviews in order to clarify participants’ thoughts as needed, while participants were encouraged to reiterate their responses in case of any uncertainty.	17 nurses.
19. Başoğul, C. (2020). Conflict management and teamwork in workplace from the perspective of nurses. *Perspectives in Psychiatric Care,* 57, 610–619. [40]	The aim was to explore the relationship between the conflict management strategies utilized by nurses and their attitudes towards teamwork.	The study was structured as a descriptive, cross-sectional, and relational investigation. Three instruments were employed for data collection: a sociodemographic questionnaire, the TeamSTEPPS Teamwork Attitudes Questionnaire (T-TAQ), which included 5 sub-scales, and the Rahim Organizational Conflict Inventory-II (ROCI-II).	228 nurses.
20. Ovčina, A., Begić, S., Eminović, E., Hrapović, E., Marjanović, M., Dido, V., Konjo, H. and Šljivo, E. (2023). Conflict situation management in nursing clinical practice. *The Journal of Health Sciences and Medicine*, 9(1), 24-31. [41]	The aim was to explore the possible link between conflict occurrences in nursing clinical practice and nurses’ discontent with their work environment, the lack of motivating factors, insufficient team communication, and the hierarchical dynamics between superiors and subordinates.	The study employed descriptive, analytical, and comparative methods, by utilizing induction, deduction, and compilation. The questionnaire developed by the original author through a review of scientific literature and practical evidence constituted the study instrument.	146 nurses.
21. Akel, D. T. and Elazeem, H. A. (2015). Nurses and physicians’ point of view regarding causes of conflict between them and resolution strategies used. *Clinical Nursing Studies*, 3(4), 112–120. [42]	The study aimed to investigate and compare the viewpoints of nurses and physicians regarding the causes of conflicts and the strategies used to resolve them.	The study employed an analytical cross-sectional design. A convenience sampling technique was utilized to enroll eligible nurses and physicians. The research instruments included a self-administered questionnaire with sections for demographic information of both nurses and physicians, along with two scales: the Conflict Causes Questionnaire and the Thomas–Kilmann Conflict Mode Instrument (TKI).	Out of 271 participants, 132 were nurses.
22. Dewi, R. R. C., Yanti, N. E. D., Rahajeng, I. M. and Krisnawati, K. M. S. (2022). Nurses’ Perceptions About Conflict Management Strategy of Head Nurse. *Nursing and Health Sciences Journal,* 2(3), 224–227. [43]	The study aimed to depict nurses’ perceptions of their head nurses and the strategies typically utilized for conflict management in their professional context.	This research employed a descriptive quantitative approach with a cross-sectional design. Purposive sampling was utilized to select participants. Data collection involved validated conflict management questionnaires. Univariate analysis was conducted to analyze the data.	71 nurses from a hospital in Bali.
